# Card games are effective tools to enhance foundation year health and safety inductions

**DOI:** 10.1002/2211-5463.70140

**Published:** 2025-10-13

**Authors:** Rumana Rafiq, Holly Matthews, Daniya Abedalreza, Faten Yahya, Matthew Allan Jones

**Affiliations:** ^1^ Biomedical Research Centre University of Salford Manchester UK; ^2^ School of Science, Engineering and Environment University of Salford Manchester UK

**Keywords:** card games, foundation year, health and safety, higher education, laboratory education, playful learning

## Abstract

Health and safety knowledge is critical in a laboratory setting but is often taught passively. As passive learning approaches are associated with lower student engagement and subsequent attainment, higher education institutions are increasingly focussed on active learning methodologies. One such approach is the use of card‐based games to gamify learning. Based on this, this study designed and evaluated three health and safety card games focussing on personal protective equipment, hazard symbols and laboratory equipment identification. Students' health and safety knowledge was evaluated using a 10‐point scale before and after completion of all three games. Wider pedagogical impact on factors such as student experience, transferable skill development and gamified learning value was also evaluated using open‐answer questions or a 5‐point Likert scale. A total of 91 foundation year students participated in the study. Most students reported positive responses regarding their enjoyment (89.9 %) and learning (74.7 %) from the games. Students liked their design (80.1 – 91.1%) and visual appeal (82.3 – 91.1 %), considering them a valuable addition to their laboratory induction (89.9 %). Pre‐ and postevaluation revealed a significant increase in self‐perceived knowledge of health and safety (6.4 ± 2.1 to 8.4 ± 1.5, *P* < 0.001), PPE (7.6 ± 2.0 to 8.7 ± 1.5, *P* < 0.001), laboratory hazards (6.8 ± 1.9 to 8.2 ± 1.5, *P* < 0.001) and laboratory equipment (6.3 ± 2.1 to 8.2 ± 1.8, *P* < 0.001). Stratification of participants based on socio‐economic factors and university entry qualifications revealed no significant differences. These findings highlight the wide benefits of card games as active learning tools to enhance health and safety education while providing a positive and equitable student experience.

## Graphical abstract

A series of three card games was developed to actively deliver health and safety education to foundation year students. Most students reported positive responses regarding their enjoyment (89.9 %) and learning (74.7 %) from all games evaluated. Data revealed significant improvements in health and safety knowledge (*P* < 0.0001). All games were equitable to all students when stratified on socio‐economic factors.

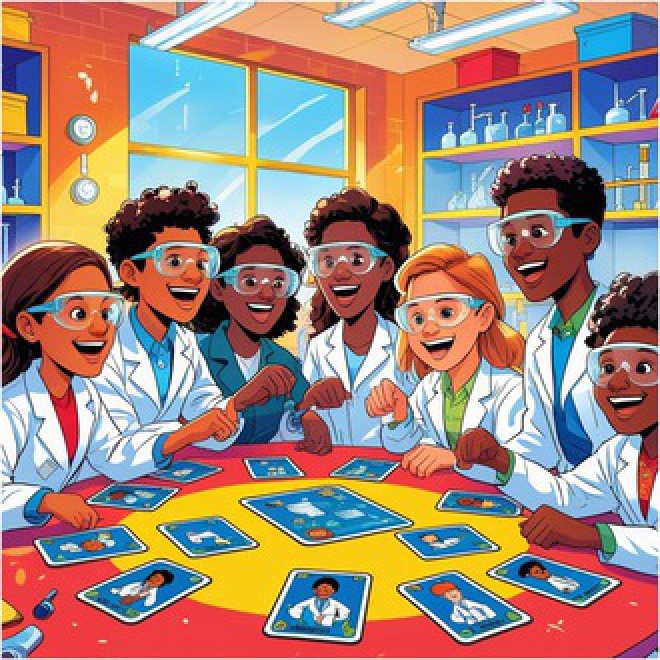

**Abbreviations**
ANOVA: analysis of variance
COSHH: control of substances hazardous to health
GHS: global harmonised system of classification
H&S: health and safety
HCPC: health and care professions council
HE: higher education
PPE: personal protective equipment
TUNDRA LSOA: tracking underrepresentation by area lower super output area

Foundation years are preundergraduate programmes designed to help students develop the skills and knowledge required for successful progression to an undergraduate degree [1, 2]. Their purpose is to increase access to higher education within the United Kingdom, offering the opportunity for students to transition to different subject areas, enter higher education as a mature student or provide an extra year of study to catch up with their peers [3, 4]. Over the past decade, enrolment in foundation year programmes has increased significantly. According to the Higher Education Policy Institute, there has been over an eightfold rise in enrolments, from 8470 in 2011/2012 to 69 325 in 2021/2022 [5, 6]. However, despite this rapid rise in demand and their role in supporting social mobility via improving access to higher education, they are often seen as ‘feeder’ programmes for undergraduate‐level study or an additional source of revenue for higher education institutes. There also remain concerns in some academic circles surrounding their delivery methodology, lack of consistency across institutions and the transition from foundation year to undergraduate programme [5]. The latter has also been highlighted by students as a potential barrier to continuation into further higher education study [7].

Due to the widening participation remit of foundation years, many students enrolling in bioscience foundation programmes come from nontraditional backgrounds and may have had little or no prior exposure to laboratory environments, let alone an understanding of health and safety protocols. It is therefore essential to provide appropriate scaffolding to support their transition, and to avoid assuming they possess prior bioscience experience simply because they are enrolled in a bioscience foundation year. Academic staff should also consider their learning approaches to ensure they are equitable and suitable for students of all backgrounds and experience levels. This is particularly important for foundation year learners due to the diverse backgrounds from which they enter higher education. As such, disseminating the knowledge of laboratory health and safety is important for these students as they may not have previous exposure, unlike peers who have studied subjects including biology, chemistry and physics, where health and safety is taught at a high school/college (ages 11–18 years) level. Further to this, a large proportion of foundation year students may be from international backgrounds where health and safety practices may vary, and they may not be exposed to the same level of health and safety knowledge.

Maintaining health and safety within laboratory environments is a fundamental priority for all laboratory‐based disciplines due to legal, student safety and learning requirements. The legal framework of laboratory health and safety is overseen in the United Kingdom by numerous pieces of legislation, including the Health and Safety in the Workplace Act 1974 and the Control of Substances Hazardous to Health (COSHH) Act 2002, with equivalent legislation observed in the majority of countries around the world [8]. To this end, ensuring that all students acquire health and safety knowledge is imperative and that they can evidence this knowledge before entering the laboratory for taught classes is vital for maintaining compliance with these regulations.

Health and safety is typically introduced during laboratory inductions, in which key concepts are explained to students through traditional lectures, with comprehension assessed via a quiz or test [9]. Some practitioners now incorporate problem‐based learning elements to support the real‐world transfer of this learning [10]. However, it has been shown that modern students do not engage fully with more traditional dissemination approaches such as lectures and are more receptive to pedagogical approaches that include interaction and the application of knowledge [11, 12, 13].

This is where active and gamified learning approaches may offer a valuable means of delivering health and safety knowledge more effectively. Gamified learning seeks to employ game principles and apply them to educational content with the aim of creating immersive and engaging learning opportunities [11, 14, 15, 16]. Numerous studies have shown that gamified learning experiences support student knowledge acquisition [17, 18, 19], maintain student engagement [20, 21] and enjoyment [22, 23, 24], and foster transferable skill development [25]. There are numerous methods through which gamified learning can be achieved; for example, the development of video games [26], escape rooms [18], interactive quizzes [21], board games [27] and card games [28]. The latter of these would be preferred for laboratory inductions due to their stand‐alone, physical, analogue nature and ease of delivery within a laboratory environment, where the use of certain technologies (mobile devices, laptops, virtual reality headsets, etc.) may be restricted due to health and safety regulations.

Card games have the advantage of being significantly quicker to complete than board games, allowing students to play multiple rounds in a single session. This repetition can enhance engagement and reinforce learning more effectively. Card games have been shown in numerous studies to have positive pedagogical benefits across a range of subject areas in enhancing knowledge [28, 29, 30, 31], increasing engagement and enjoyment of learning content [29, 32, 33, 34] and transferable skill development [35, 36, 37]. However, despite these benefits, there is limited evidence on the use of these approaches with foundation year students and no documented instances of their application in health and safety education.

Based on this, this study sought to evaluate a series of card games for integration as a part of laboratory health and safety inductions and determine their pedagogical impact in enhancing foundation year student experience, skill development, and health and safety knowledge.

## Materials and methods

### Participants demographics

The participants for this study were recruited from the bioscience foundation year at the University of Salford during the induction to the 2024–2025 academic year. This Level 3 equivalent HE entry programme offers students an opportunity to access HE to reskill or reattempt Level 3 qualifications before progressing into Level 4 undergraduate programmes including Biomedical Science, Human Biology and Infectious Diseases, Biochemistry, Pharmaceutical Science, Biological Sciences, Wildlife Conservation, Marine Biology, Zoology and Geography. Of the 123 individuals enrolled within the bioscience foundation year in the academic year 2024 – 2025, 84 participants completed the overall survey (79 consented for inclusion), and 92 individuals completed the pre‐ and postsurvey (91 consented for inclusion). The demographic data for all session participants who consented for the overall survey, which contained demographic data (*n* = 79), are summarised in Table 1.

**Table 1:** Health and Safety card game session participant demographics.

Demographic	
Age (years)	19.5 ± 4.2
Gender (% Female)	58.2 %
Minoritised ethnicity (%)	46.8 %
Home or international (% Home)	98.7 %
Multiple deprivation decile	3.8 ± 3.0
POLAR4	2.7 ± 1.4
TUNDRA LSOA	3.0 ± 1.3
ADULT HE	2.4 ± 1.3
Entry qualification	A‐level (56 %), BTEC (30 %), Access course (5 %), Other (9 %)
Foundation year programme of study	Biomedical Science (33 %), Human Biology and Infectious Diseases (6 %), Biochemistry (6 %), Pharmaceutical Science (16 %), Biological Science (8 %), Wildlife and Zoo Biology (5 %), Wildlife Conservation (8 %), Marine Biology (4 %), Zoology (9 %), Geography (5 %)

The studies involving human participants were reviewed and approved by the Ethics Committee at the University of Salford under the Ethics Application ID: 1035. All questionnaires and analysis procedures were also approved by this ethics committee.

### Health and safety card game design

As a part of this project, three card games were designed with the rationale of supporting student understanding in the field of laboratory health and safety, something that is often delivered passively by academic staff, and to support the transition to HE. The three card games, described in Sections ‘Health and safety Hazard hunter’, ‘Personal protective equipment (PPE) lab gear up’ and ‘Lab equipment identifier’, were designed to address the aforementioned issues.

#### Health and safety Hazard hunter

The first game Health and Safety Hazard Hunter [38] was designed to facilitate student knowledge on the Global Harmonised System of Classification (GHS) hazard symbols. The game is designed as a matching game where participants must identify the correct GHS pictogram, the associated hazard, the definition and an example chemical with the aim of providing real‐world context of where students may interact with chemicals associated with each hazard.

#### Personal protective equipment (PPE) lab gear up

The second game PPE Lab Gear Up [39] is a scenario‐based game, where students draw scenario cards from a deck and must dress an example character in the appropriate PPE for the activity described on the scenario cards. The game aims to showcase students what PPE will be available to them within a laboratory environment and to teach them the specific scenarios where modified PPE is appropriate.

#### Lab equipment identifier

The third and final game, Lab Equipment Identifier [40], is a matching pairs game designed to introduce key pieces of laboratory equipment that are routinely used within their education and future professional practice for the measurement of liquids within a laboratory environment. It also sought to showcase to students how accurate each piece of equipment was for this task. Students were tasked with drawing cards from a deck of equipment cards and matching them to their associated definitions. They were then asked to determine whether the piece of equipment was categorised as high, medium or low accuracy for liquid handling and measurement.

### Session design

The session was designed to be delivered as an alternative to a previous traditional lecture covering laboratory health and safety. The card games session was delivered within a 1‐h health and safety session conducted within the main teaching laboratory, with students asked to complete the presurvey upon entry to the session. Students were then given a brief (10 min) introduction to the laboratory and a briefing on how the session would work. Students were then given 15 min per card game (total 45 min) to play each game in groups of approximately 3 – 5. After completion of all games, students were asked to complete both the overall survey and the postsurvey to determine the impact of the card games.

### Survey design and delivery

All questions were designed as either open‐answer questions or based on a 5‐point Likert scale, with the responses categorised as scores of one or two indicating a negative response, a score of three indicating a neutral response, and scores of four or five indicating a positive response, modified from those surveys previously described in Hussain *et al*. [41] and Morgan and Jones [18] (Table S1). The questions used in the survey were distributed into multiple categories to evaluate various aspects of the health and safety card games, including health and safety knowledge acquisition, student experience and transferable skill development, game design and difficulty, and participants' backgrounds and demographics. Game‐specific questions were repeated individually for all games used as a part of this study. Students were also asked to complete a pre‐ and post survey (Table S2), which was comprised of a 10‐point scale to assess student understanding before and after the delivery of the card games. The content of the survey was validated based on face validity through subject matter experts creating the survey, and question appropriateness was tested by the target population in previously published work. Surveys were checked before delivery to ensure both content and construct validity were ensured.

Key metrics of HE access and participation used within previous studies [41, 42] including higher education participation (POLAR4) [43], tracking underrepresentation by area lower super output area (TUNDRA LSOA) [44], ADULT HE [45] (all scored based on a 1–5 scale, with 1 being low and 5 being high) and the United Kingdom Government Ministry of Housing, Communities and Local Government English indices of deprivation (https://imd‐by‐postcode.opendatacommunities.org/imd/2019) (scored on a scale of 1–10, with 1 being high deprivation and 10 being low deprivation) were used to stratify participants based on their backgrounds and likelihood to access HE, alongside other demographic data associated with HE awarding gaps within the United Kingdom. These metrics are utilised to determine the efficacy of pedagogical activities in cohorts of high and low higher education access to determine their equity.

### Statistical analysis

All statistical analysis was performed using graphpad prism version 10.4.0 (GraphPad Software, Boston, MA, USA). Data normality was evaluated using the Shapiro–Wilk test or Kolmogorov–Smirnov test for normality, depending on the sample size. All two‐group comparisons were conducted using parametric paired *t*‐tests. All multigroup comparisons were performed through analysis of variance (ANOVA) followed by pairwise comparisons using a Tukey *post hoc* test. All data are expressed as mean ± standard deviation. Statistical significance was set at *P* ≤ 0.05.

## Results

### Health and safety card games produce a positive effect on foundation year student experience and perceived transferable skills development

**Fig. 1:**
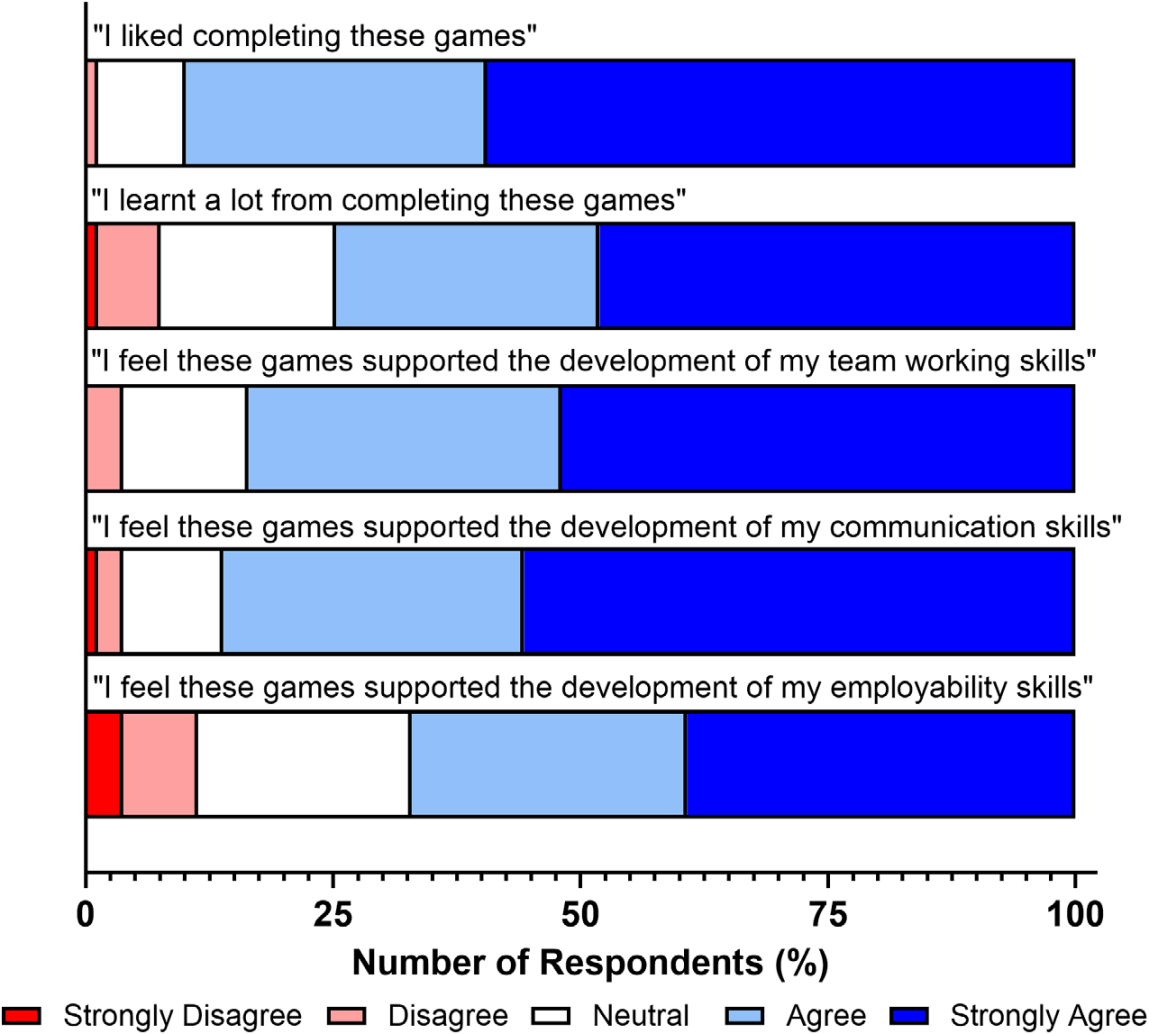
Health and safety card games produce a positive effect on foundation year student experience, knowledge acquisition, and perceived transferable skills development. Shades of blue indicate positive responses, white indicates a neutral response, and shades of red indicate negative responses. *n* = 79 for all bars.

Student questionnaire responses relating to student experience and transferable skill development are shown in Fig. 1. These responses are highly positive, with 89.9 % of students reporting an enjoyment of completing these games, with 74.7 % stating that they learned a lot through the completion of these active H&S educational resources. Evaluation of students' self‐reported transferable skill development revealed similarly positive outcomes. A majority reported improvements in teamwork (83.5 %) and communication skills (86.1 %), attributed to the session's pedagogical design outlined in Section ‘Session design’. However, a lower proportion (67.1 %) noted a positive impact on their general employability skills, suggesting that more specific skills were more clearly recognised by students.

### Health and safety card games have a positive impact on students perceived health and safety knowledge

**Fig. 2:**
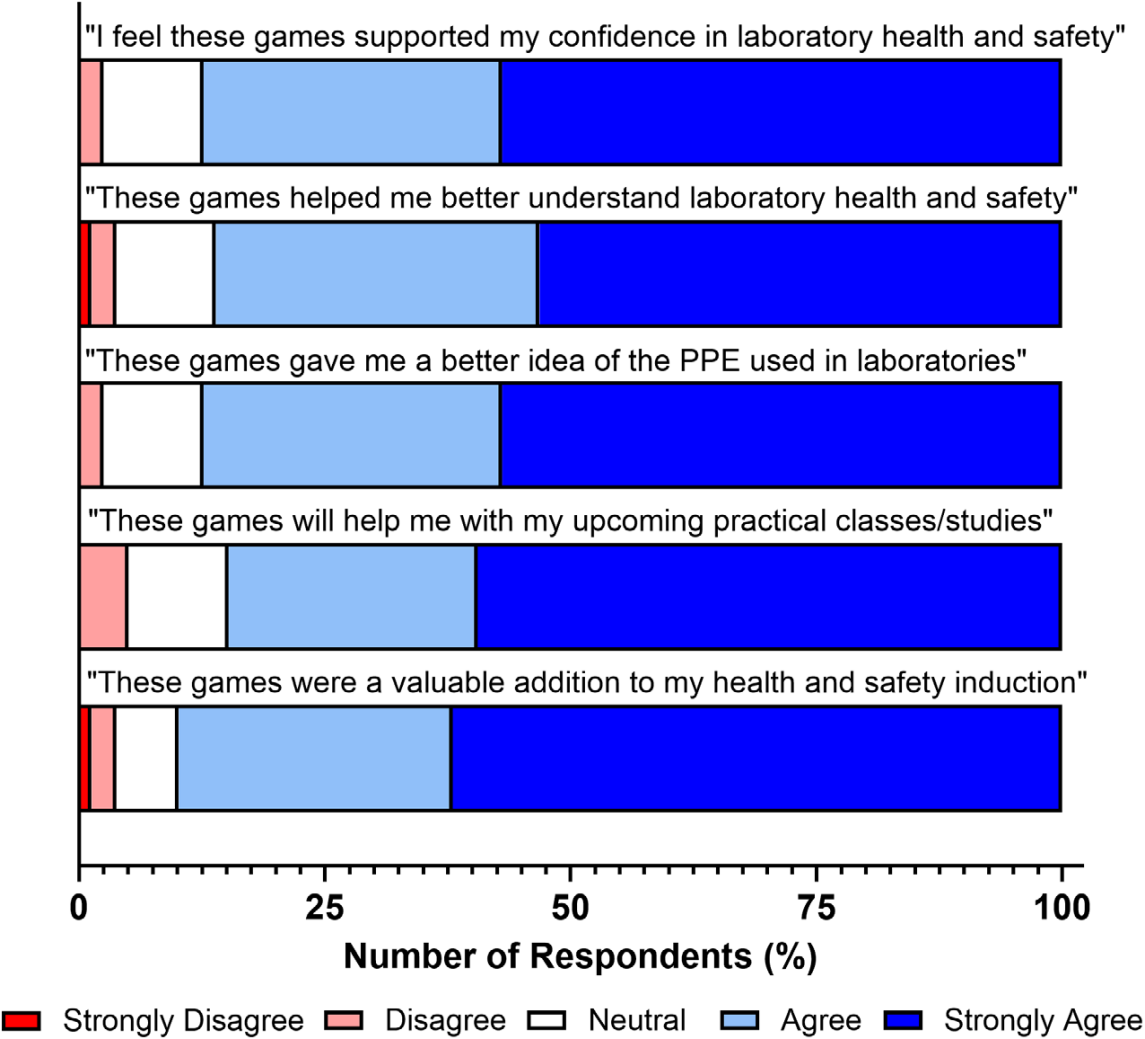
Health and safety card games have a positive impact on students' perceived health and safety knowledge. Shades of blue indicate positive responses, white indicates a neutral response, and shades of red indicate negative responses. *n* = 79 for all bars.

Overall evaluation of students' perceived health and safety knowledge following the completion of the card games session again highlighted the positive impact of this approach as a methodology for delivering H&S content (Fig. 2). Participants reported that playing these games enhanced their confidence in laboratory health and safety (87.3 %) and allowed them to better understand laboratory H&S (86.1 %) and the role of PPE within laboratories (87.3 %). The majority of students also reported the use of card games for health and safety education to be helpful for their upcoming practical studies (84.8 %) and considered them a valuable addition to laboratory health and safety inductions (89.9 %).

### Individual health and safety card games are effective tools for enhancing student experience and health and safety knowledge within laboratory inductions

**Fig. 3:**
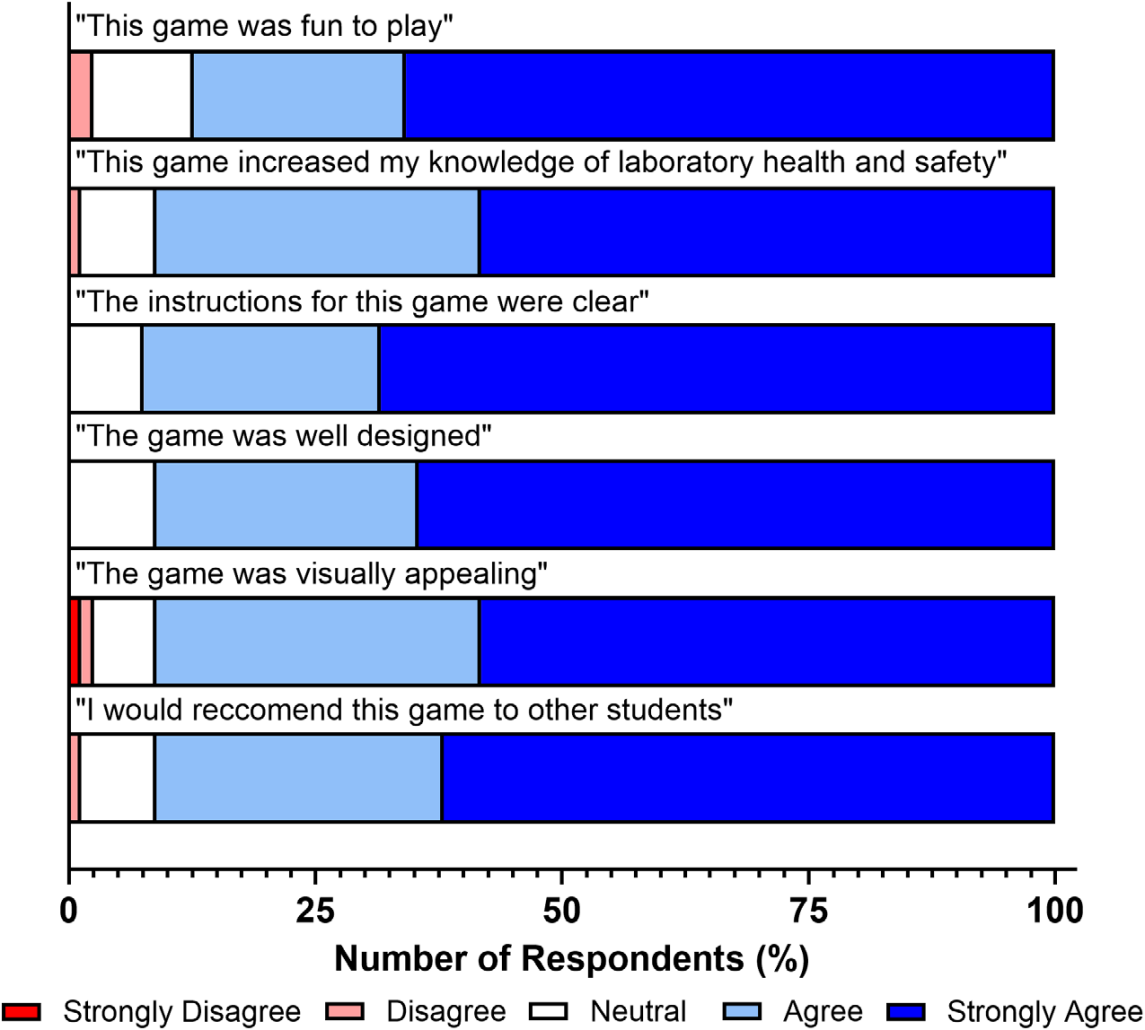
Student feedback on the Health and Safety Hazard Hunter card game. Students playing the Health and Safety Hazard Hunter card game reported positive impacts on their experience, health and safety knowledge acquisition, and gameplay design. Shades of blue indicate positive responses, white indicates a neutral response, and shades of red indicate negative responses. *n* = 79 for all bars.

**Fig. 4:**
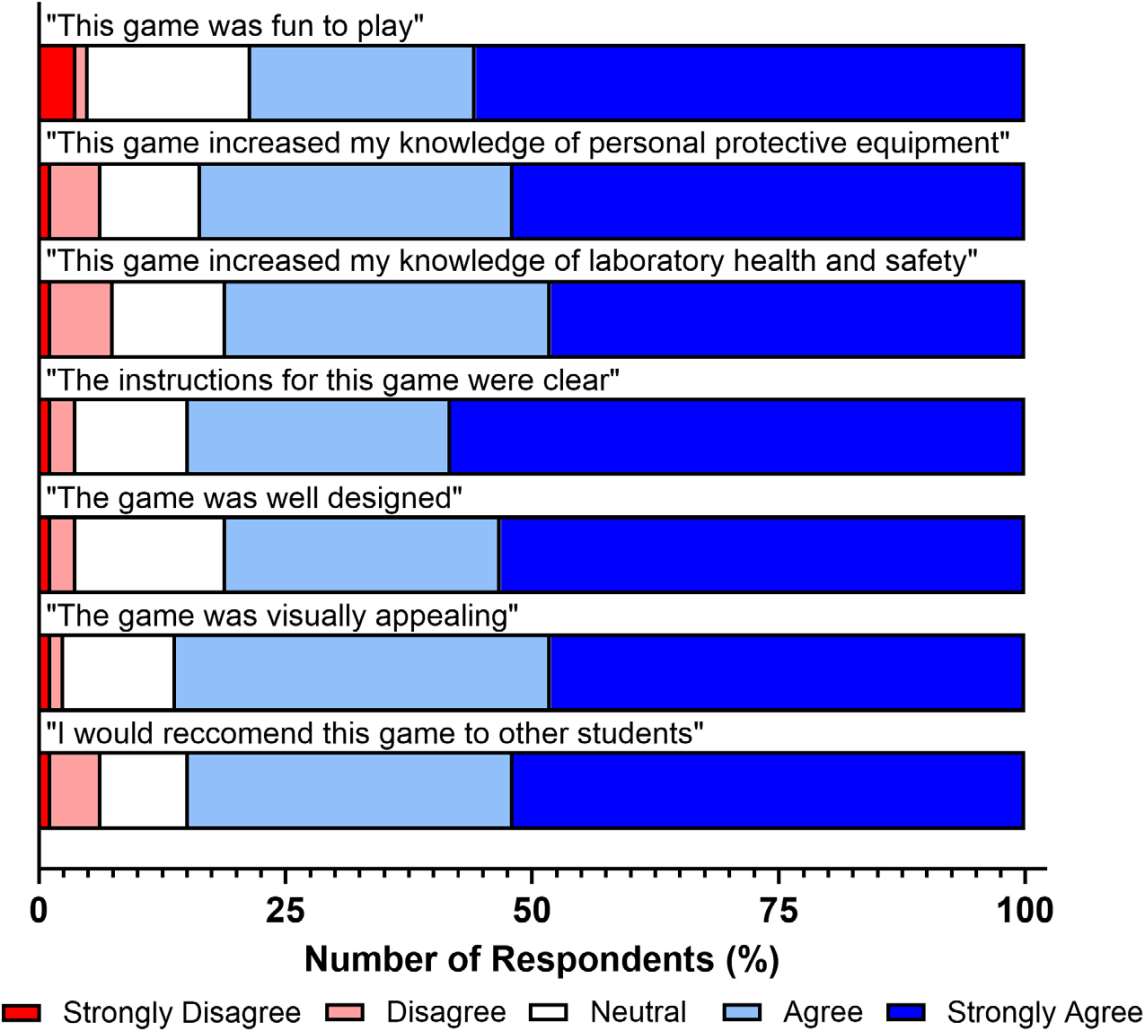
Student feedback on the PPE Lab Gear Up card game. Students playing the PPE Lab Gear Up card game reported positive impacts on their experience, health and safety knowledge acquisition, and gameplay design. Shades of blue indicate positive responses, white indicates a neutral response, and shades of red indicate negative responses. *n* = 79 for all bars.

**Fig. 5:**
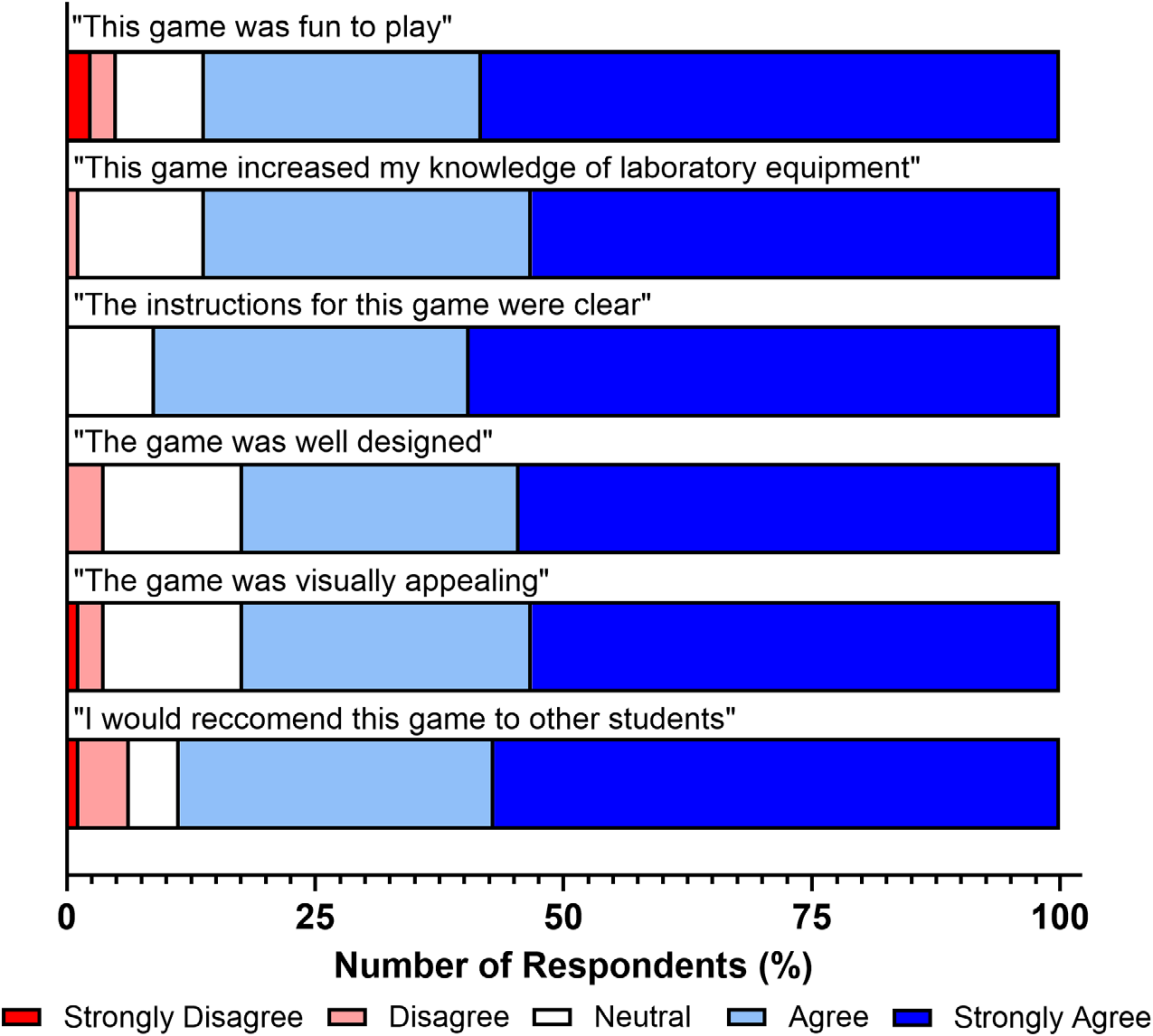
Student feedback on the Lab Equipment Identifier card game. Students playing the Lab Equipment Identifier card game reported positive impacts on their experience, laboratory equipment knowledge acquisition, and gameplay design. Shades of blue indicate positive responses, white indicates a neutral response, and shades of red indicate negative responses. *n* = 79 for all bars.

While overall evaluations of the use of card games were strongly positive (Figs 1 and 2), we aimed to determine whether the specific games used influenced students' experiences and knowledge outcomes, and whether any disparities existed between the games developed to support health and safety education. The findings of this approach are visualised in Figs 3, 4, 5.

The first of these games, Health and Safety Hazard Hunter, revealed positive impacts across all areas evaluated (Fig. 3). It was found that the majority of students reported positive experiences relating to enjoyability (87.3 %) and knowledge acquisition (91.1 %). Students also reported strongly positive experiences with core gameplay elements associated with this game, including clarity of instruction (92.4 %), game design (91.1 %) and visual appeal (91.1 %). Students (91.1 %) additionally stated that they would recommend the use of this game to their peers to enhance health and safety inductions.

The second of these games, Personal Protective Equipment (PPE) Lab Gear Up, also received positive feedback from students (Fig. 4). Student responses showed this game to be fun to play (78.5 %), enhanced their health and safety knowledge (81.0 %), and increased their knowledge of personal protective equipment (83.5 %). Students also reported positive responses relating to game design, with 81.0 % stating the game was well designed, with clear instructions (84.8 %) and visual appeal (86.1 %). Most students (84.8 %) said they would recommend this game to fellow students to enhance knowledge of personal protective equipment.

The final game evaluated, Lab Equipment Identifier, received feedback consistent with the other two games. A majority of students reported it as enjoyable (86.1 %) and effective in increasing their knowledge of laboratory equipment (86.1 %). As with the other games, positive responses were also recorded for clarity of instructions (91.1 %), game design (82.3 %) and visual appeal (82.3 %). Additionally, 88.6 % of students stated they would recommend the game to others. Comparisons between each of the games revealed no significant difference in pedagogical impact for all games evaluated in this study.

### Card games significantly enhance students perceived knowledge acquisition within laboratory inductions

We next sought to determine whether the utilisation of card games as a part of health and safety induction enhanced perceived knowledge acquisition. To do this, a pre–post survey was conducted to measure knowledge before the commencement of the induction and following the completion of the session.

**Fig. 6:**
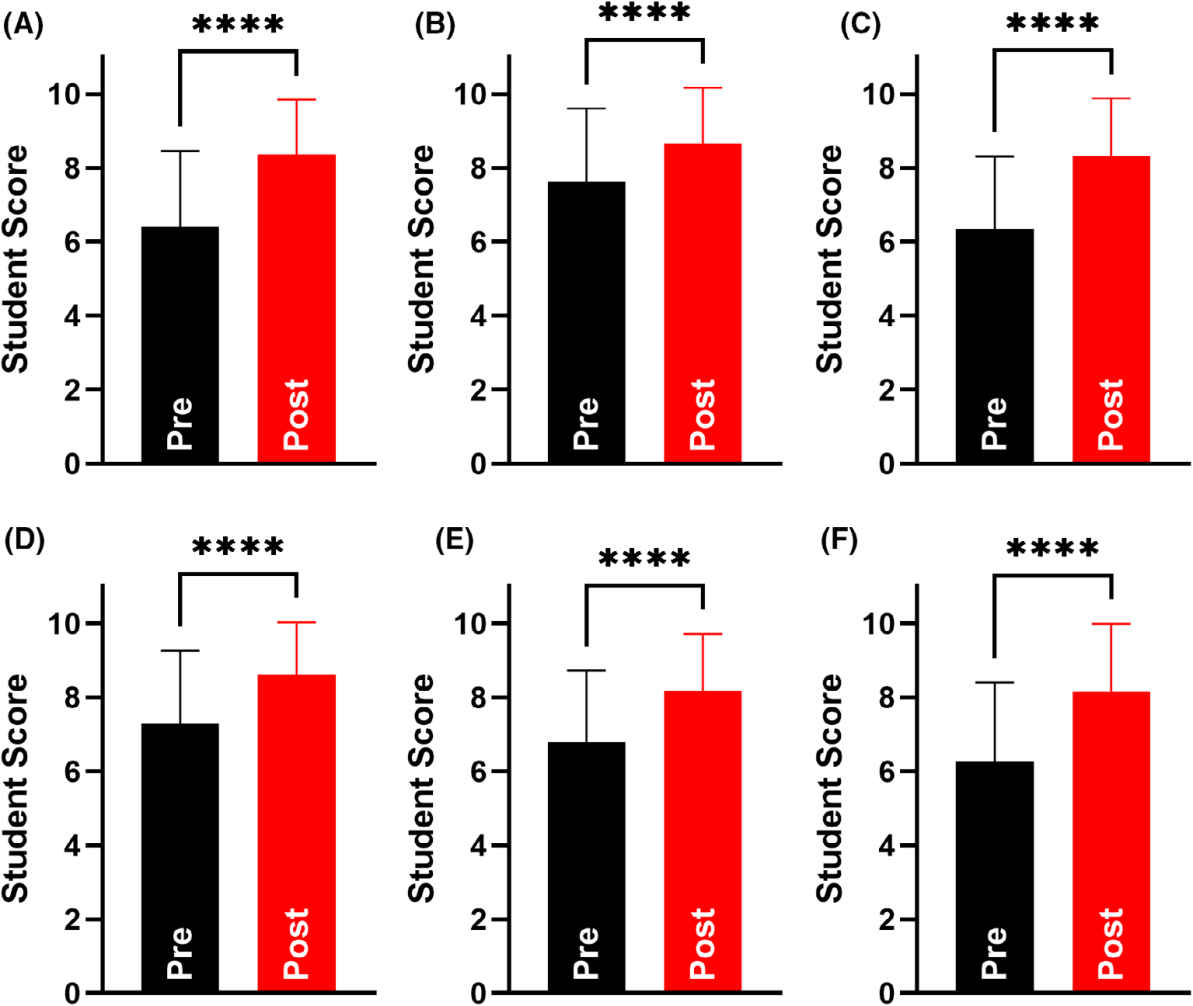
The utilisation of card games within health and safety inductions significantly enhances students perceived knowledge acquisition. Students were asked to evaluate their (A) Health and Safety knowledge, (B) personal protective equipment knowledge, (C) health and safety symbol knowledge, (D) when PPE should be worn within the laboratory, (E) identification of laboratory risks, and (F) identification of key laboratory equipment. Black bars indicate student score before the induction session (Pre) and red bars indicate student score after completion of the induction session (Post). *n* = 91 for all panels. **** indicates statistical significance to a *P* value of < 0.0001 following a paired *t*‐test. All data expressed as mean ± standard deviation.

Student evaluations (*n* = 91) revealed a significant increase in perceived knowledge across all areas analysed (Fig. 6). Significant increases in perceived knowledge were identified relating to Health and Safety knowledge (Pre: 6.4 ± 2.1, Post: 8.4 ± 1.5, *P* < 0.0001, Fig. 6A), personal protective equipment (Pre: 7.6 ± 2.0, Post: 8.7 ± 1.5, *P* < 0.0001, Fig. 6B), health and safety symbols (Pre: 6.3 ± 2.0, Post: 8.3 ± 1.6, *P* < 0.0001, Fig. 6C), when PPE should be worn within the laboratory (Pre: 7.3 ± 2.0, Post: 8.6 ± 1.4, *P* < 0.0001, Fig. 6D), identification of laboratory risks (Pre: 6.8 ± 1.9, Post: 8.2 ± 1.5, *P* < 0.0001, Fig. 6E) and the identification of key laboratory equipment (Pre: 6.3 ± 2.1, Post: 8.2 ± 1.8, *P* < 0.0001, Fig. 6F).

### Are health and safety card games equitable tools to enhance laboratory health and safety education?

In higher education, it is essential to ensure that all teaching and learning approaches are equitable for all students, with the goal of addressing and preventing awarding gaps that have emerged over the past decade, particularly those linked to minority ethnic status, socio‐economic status and higher education entry qualifications. Based on this, we sought to determine whether the three health and safety card games elicited equitable responses when stratified by factors commonly associated with awarding gaps in higher education.

**Fig. 7:**
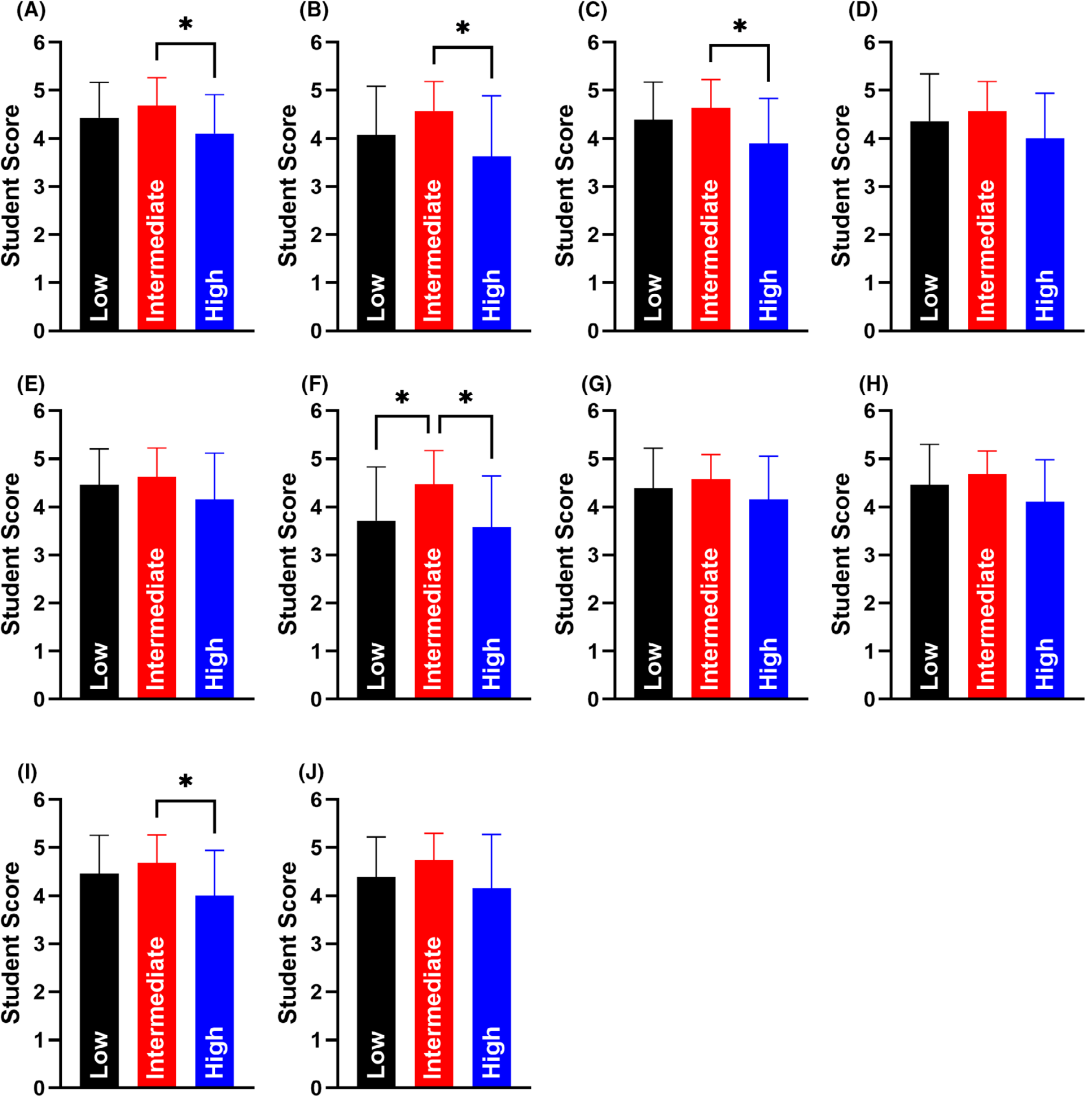
Student responses following completion of health and safety card games stratified on their likelihood to participate in higher education (POLAR4). Student questions relating to (A) enjoyment, (B) learning, (C) teamwork development, (D) communication development, (E) health and safety confidence, (F) employability development, (G) health and safety understanding, (H) personal protective equipment knowledge, (I) support upcoming classes, and (J) valuable addition to laboratory health and safety inductions were stratified. Black bars indicate student scores from low POLAR4 areas (POLAR4 scores of 1 or 2) (*n* = 28), red bars indicate student scores from intermediate POLAR4 areas (POLAR4 score of 3) (*n* = 19), and blue bars indicate student scores from high POLAR4 areas (POLAR4 scores of 4 or 5) (*n* = 19). *Indicates statistical significance to a *P* value of < 0.05 following a one‐way ANOVA with a Tukey *post‐hoc* test. All data expressed as mean ± standard deviation.

When participants were stratified according to their higher education participation levels using POLAR4, no significant differences were observed between those from low (scores of 1 and 2) and high (scores of 4 and 5) participation backgrounds (Fig. 7). However, significant differences emerged between participants from intermediate (score of 3) and those from both low and high POLAR4 groups. Significant decreases in average response score were seen when participants from intermediate and high POLAR4 backgrounds responded to questions relating to enjoyment (Intermediate: 4.68 ± 0.58, *n* = 19, High: 4.11 ± 0.81, *n* = 19, *P* = 0.042, Fig. 7A), learning (Intermediate: 4.58 ± 0.61, *n* = 19, High: 3.63 ± 1.26, *n* = 19, *P* = 0.013, Fig. 7B), teamwork development (Intermediate: 4.63 ± 0.60, *n* = 19, High: 3.89 ± 0.94, *n* = 19, *P* = 0.014, Fig. 7C), employability development (Intermediate: 4.47 ± 0.70, *n* = 19, High: 3.58 ± 1.07, *n* = 19, *P* = 0.021, Fig. 7F) and their belief it would support upcoming classes (Intermediate: 4.68 ± 0.58, *n* = 19, High: 4.00 ± 0.94, *n* = 19, *P* = 0.025, Fig. 7I). When stratification was conducted, we also observed a significantly lower score in low POLAR individuals compared to intermediate participants only in their responses relating to employability development (Intermediate: 4.47 ± 0.70, *n* = 19, Low: 3.71 ± 1.12, *n* = 28, *P* = 0.035, Fig. 7F). All other comparisons between low and intermediate participants were found to be non‐significant.

When participants were stratified by TUNDRA LSOA (Fig. S1), ADULTHE (Fig. S2) and highest qualification on entry to higher education (Fig. S3), gender (Fig. S4) and ethnic status (Fig. S5), no significant differences were observed for any of the questions when stratified based on these parameters associated with higher education awarding gaps within the United Kingdom.

### Subject group analysis

**Fig. 8:**
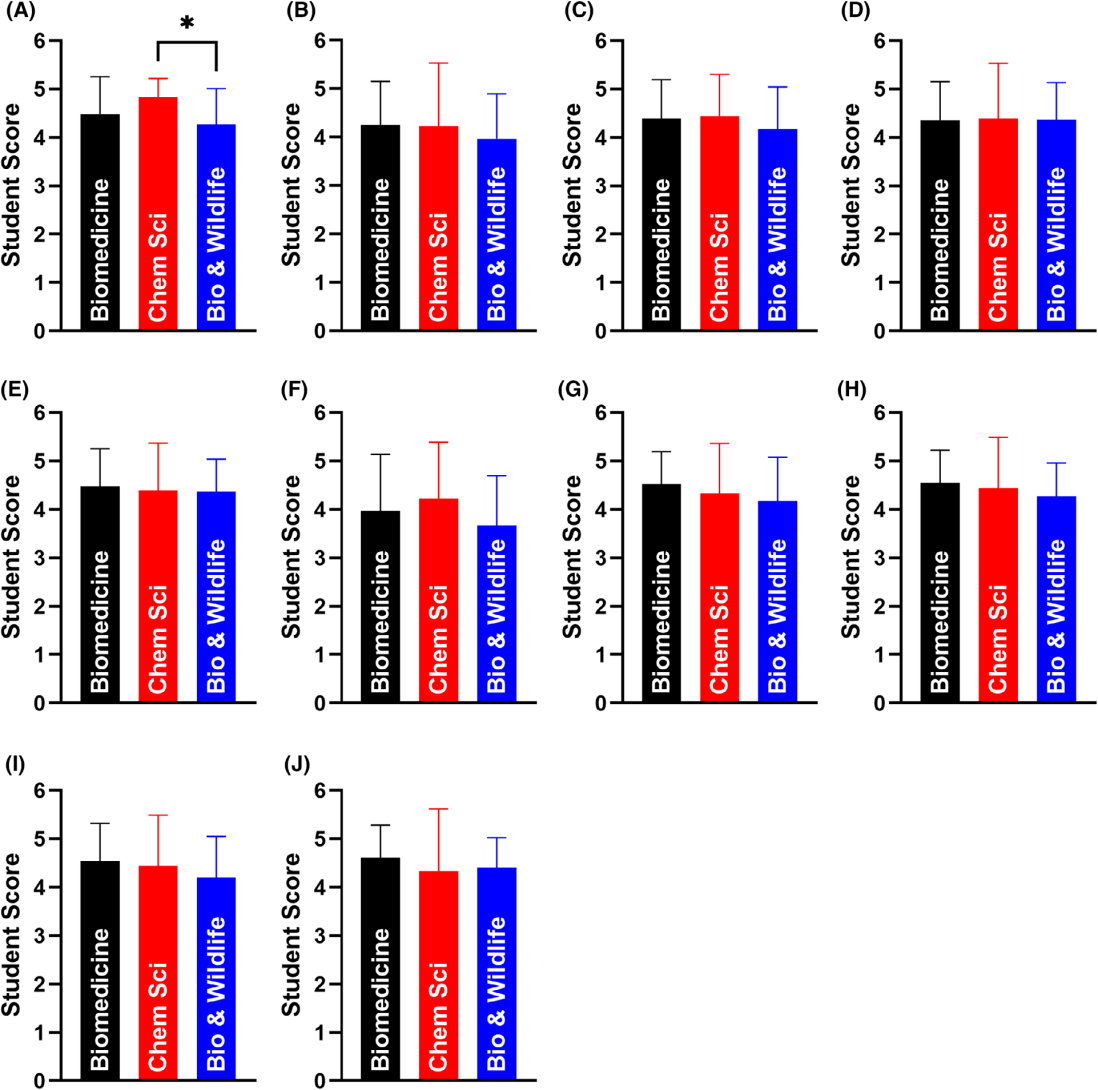
Student responses following completion of health and safety card games stratified on their foundation year programme type. Student questions relating to (A) enjoyment, (B) learning, (C) teamwork development, (D) communication development, (E) health and safety confidence, (F) employability development, (G) health and safety understanding, (H) personal protective equipment knowledge, (I) support upcoming classes, and (J) valuable addition to laboratory health and safety inductions were stratified. Black bars indicate student scores from biomedicine programmes (*n* = 31), red bars indicate student scores from chemical science programmes (*n* = 18), and blue bars indicate student scores from biology and wildlife programmes (*n* = 30). *Indicates statistical significance to a *P* value of < 0.05 following a one‐way ANOVA with a Tukey *post‐hoc* test. All data expressed as mean ± standard deviation.

Given the diverse range of programmes that include foundation years, it was important to assess whether the health and safety card games were equitable across the different programme types or if they disproportionately benefited certain groups. To investigate this, participants were stratified into three categories: biomedicine (Biomedical Science and Human Biology and infectious diseases), chemical sciences (Biochemistry and Pharmaceutical science) and Biology and Wildlife (Biological science, Geography, Marine biology, Wildlife and Zoo Biology, Wildlife Conservation, and Zoology). The findings of this stratification are visualised in Fig. 8.

Overall, we observed minimal difference in average response score within all three groups for most questions evaluated (Fig. 8B–J). However, when the question relating to enjoyment was stratified, there was a significantly lower response score from biology and wildlife students compared to those studying chemical science‐based foundation years (Chemical Science: 4.83 ± 0.38, *n* = 18, Biology and Wildlife: 4.27 ± 0.74, *n* = 30, *P* = 0.020, Fig. 8A). All other comparisons produced no significant difference between subject groups.

## Discussion

Developing laboratory health and safety knowledge is a critical first step in degree programmes that involve laboratory work. This is particularly important for foundation year students, who often come from educational backgrounds with limited or no prior exposure to laboratory environments. Such variability is a result of widening participation initiatives and alternative entry requirements typical of foundation‐level programmes, which differ from standard undergraduate entry routes. Based on this, we sought to determine whether an active gamified approach to teaching laboratory health and safety was a viable alternative to traditional practice, through the evaluation of the authors' previously designed card games series [38, 39, 40] to address key threshold concepts in laboratory health and safety. Our study demonstrates that the creation of three card games provides an enjoyable active gamified learning experience for foundation year students (Fig. 1), while also enhancing their perceived knowledge of laboratory health and safety (Figs 2 and 6). This approach is equitable and suitable for the diverse range of students [38, 39, 40] (Fig. 7) and subject areas (Fig. 8) typically entering higher education through the foundation year route.

Health and safety knowledge is paramount in the laboratory, not just to maintain the safety and wellbeing of both students and academic staff during laboratory class delivery, but also due to the legal framework, which underpins health and safety in the workplace [9]. It is therefore important that students learn and are able to apply this skill within the context of future employment. Indeed, the majority of job specifications across a wide range of disciplines emphasise knowledge and competency in health and safety as essential, with noncompliance in the workplace potentially resulting in disciplinary action or termination of employment.

For example, the Health and Care Professions Council (HCPC), which regulates the Biomedical science profession within the United Kingdom, outlines in its standards of proficiency for Biomedical Scientists [46] the importance of health and safety. Specifically, Standard 14 states that biomedical scientists must ‘understand the need to maintain the safety of themself and others’, ‘select appropriate personal protective equipment and use it correctly’ and ‘establish safe environments for practice, which appropriately manage risk’. This highlights the critical role of health and safety in professional practice. Similar requirements are echoed across other disciplines that include laboratory work. Health and safety is a core learning outcome mandated by numerous degree accrediting bodies, including the Royal Society of Biology [47], the Royal Society of Chemistry [48] and the Institute of Biomedical Science [49]. These expectations reinforce the idea that health and safety knowledge should form an essential part of the initial curriculum for all learners. This ensures they are adequately prepared for the workplace and able to demonstrate their competence in job applications and interviews.

Our study highlights the potential of this approach as a pedagogical tool across the biological and chemical sciences, particularly through its ability to enhance students' perceived knowledge of health and safety (Figs 2 and 6). Notably, it significantly improves students' understanding of when PPE should be worn (Fig. 6D) and how to identify laboratory risks (Fig. 6E). These findings align with previous research demonstrating the effectiveness of card games in enhancing knowledge acquisition within student cohorts of biological and chemical science students from the Philippines, the United States and Mexico [31, 50, 51]. It also corroborates the findings of Steele *et al*. [52] who also reported the majority of participants were more familiar with laboratory health and safety items and equipment as a result of card game activities. While this study did not directly assess knowledge acquisition through gradable measures (since all students are required to achieve 100% on a mandatory health and safety assessment before entering the laboratory), there has been an anecdotal decrease in reported health and safety breaches following the introduction of these gamified sessions, compared to previous years using a more traditional teaching approach. However, further evaluation and longitudinal data are required to substantiate this anecdotal evidence and fully assess the long‐term impact of this intervention.

When designing card games for educational purposes, it is essential to ensure that the intended learning content is effectively conveyed and that students clearly understand how to play and complete the game. This underscores the importance of strong constructive alignment and clear instructions to support a smooth and effective learning experience. A recent meta‐analysis by Kordaki and Gousiou [30] identified clarity of instructions as a key factor valued by students, enabling them to focus on gameplay without anxiety about making mistakes. Our findings support this, with the majority of students (83.5–92.4 %) reporting that the instructions were clear (Figs 3, 4, 5). This likely contributed to their high levels of reported enjoyment and perceived knowledge acquisition. The simplicity of the card game designs further supports this outcome, as each of the three evaluated card games contained no more than eight instructional steps. This minimalist approach reduces confusion during gameplay and provides additional benefits: It simplifies delivery for educators by requiring less instruction and empowers students to engage with the games either independently or collaboratively, as was the case in this study.

In addition to supporting knowledge transfer and enhancing the student experience, card games such as those used in this study can also contribute to the development of transferable skills. The acquisition of these skills is a key component of foundation year programmes, particularly as research has shown that widening participation students often enter higher education with lower baseline levels of graduate capital and reduced academic confidence compared to their peers [53, 54]. As such, fostering transferable skills is crucial for promoting equitable outcomes and supporting students' successful transition into undergraduate study. Our findings indicate that the majority of students reported the development of key transferable skills, including teamwork (83.5 %) and communication (86.1 %) (Fig. 2). This lower proportion of positive responses relating to key employability skills compared to knowledge and employability may have resulted due to foundation year students potentially being unable to identify these employability‐related skills within the games or alternatively could not identify the relationship between health and safety and their future employment. These outcomes were supported by the group‐based delivery of the games, which encouraged students to discuss potential answers collaboratively. This peer discussion likely also facilitated peer‐to‐peer learning within groups, a pedagogical approach that has been shown to enhance both knowledge acquisition and educational confidence in student cohorts.

In addition to supporting the development of transferable skills, the timing of the health and safety induction presents an opportunity to deliver further benefits to learners. As health and safety inductions are typically among the first activities delivered in laboratory‐based modules or during programme induction, incorporating collaborative card games during this early period can encourage social interaction among students. This interaction may foster the formation of friendships and help mitigate social isolation, an issue that is increasingly prevalent in HE [55]. By promoting early peer connections, such activities have the potential to reduce withdrawal rates in the initial weeks of the academic term and contribute positively to student mental health, both areas of growing concern within the sector [56]. Thus, the integration of socially engaging, gamified learning activities at the outset of a programme may offer both academic and pastoral benefits.

When developing new pedagogical practices, it is essential to ensure they benefit all learners, thereby promoting parity of experience and helping to prevent the emergence of awarding gaps between different student populations. Our findings indicate that when student responses were stratified by socio‐economic factors commonly associated with awarding gaps (Fig. 7), no significant differences were observed between higher‐ and lower‐socio‐economic groups. We did find that students from low POLAR4 backgrounds reported lower scores relating to employability development. This may result from these students lacking the socio‐experiential capital to identify these skills to the same level as those from higher POLAR4 backgrounds due to a lack of familial experience of identifying key graduate skills.

Additionally, we found the card games applicable across a broad range of disciplines (Fig. 8), including subjects where the relevance of laboratory health and safety may be perceived as less immediate, such as wildlife, zoology and geography. Despite this, we observed equitable responses across all subject areas, further highlighting the potential for wider adoption of this approach in both undergraduate and postgraduate programmes. While outcomes may vary if these games were implemented in different international contexts, our data suggest a strong potential for this equity of experience to transfer across geographic and institutional boundaries. Nevertheless, further multi‐institutional and cross‐cultural research would be required to validate this hypothesis.

The benefits of gamified learning for enhancing knowledge acquisition, student engagement, and learning enjoyment have been well documented in the literature [17, 18, 19, 21, 22, 23, 34]. Our study supports and corroborates these findings, demonstrating positive impacts across these metrics (Fig. 1). While gamified learning may not appeal to all students and previous studies have reported mixed outcomes [12, 25], contemporary students (many whom have grown up with gaming as part of everyday life) may be more receptive to interactive and engaging learning approaches. This is particularly relevant in light of declining on‐campus engagement, often due to external commitments such as caring responsibilities or part‐time employment. Despite its potential, a key barrier to the adoption of gamified learning is the limited time and resources available to many academic staff for the development or procurement of such materials. The games presented in this study provide a simple and accessible starting point for integrating gamified learning into the curriculum, especially in the early stages of students' academic journeys. They offer educators an opportunity to evaluate the feasibility and impact of gamified methods with minimal investment. Moreover, the structure and design of these games can serve as a transferable model for adaptation into other subject areas beyond the laboratory sciences, particularly in disciplines where health and safety are a critical concern, such as construction, engineering, and allied health professions.

Given traditional lecture formats are often used for laboratory health and safety inductions, the authors acknowledge a limitation of this study as there is no direct comparison to lecture or a more traditional approach vs this gamified approach. However, anecdotal evidence from students of higher levels of study who heard about this activity reported they would have much preferred this style of learning over their lecture experience. However, further research is required to evaluate this. It is important to acknowledge that the data presented in this study were collected from a single foundation‐level cohort within one academic year. As such, student perspectives may vary across different cohorts and years. Further longitudinal research is therefore required to determine whether the positive outcomes observed, such as enhanced engagement, enjoyment and perceived knowledge acquisition, are sustained across different levels of study and over time. Nonetheless, we believe the strong pedagogical benefits demonstrated in this study are likely to persist in future cohorts and across diverse student populations. However, longer term understanding of whether these games are suitable for neurodiverse individuals should be evaluated.

## Conclusions

Overall, this study demonstrates the value of using card games to support laboratory health and safety inductions, enhancing both student knowledge acquisition and the development of key transferable skills. Furthermore, this approach promotes equity of learning experiences and shows strong potential for adaptation across the diverse range of programmes that contribute to foundation year provision.

## Conflict of interest

The authors declare no conflict of interest.

## Author contributions

Conceptualisation, RR, HM and MAJ; methodology, RR, HM and MAJ; software, MAJ; formal analysis, RR, HM and MAJ; investigation, RR, HM, DA, FY and MAJ; resources, RR, HM and MAJ; data curation, RR, HM, DA, FY and MAJ; writing – original draft preparation, RR, HM and MAJ; writing – review and editing, RR, HM, DA, FY and MAJ; visualisation, RR, HM and MAJ; supervision, RR, HM and MAJ; project administration, RR, HM and MAJ; funding acquisition, RR, HM and MAJ. All authors have read and agreed to the published version of the manuscript.

## Supporting information


**Fig. S1.** Student responses following completion of health and safety card games stratified on their likelihood to participate in higher education (TUNDRA LSOA).
**Fig. S2.** Student responses following completion of health and safety card games stratified on their likelihood to participate in higher education (ADULT HE).
**Fig. S3.** Student responses following completion of health and safety card games stratified on their higher education entry qualification.
**Fig. S4.** Student responses following completion of health and safety card games stratified on their identified gender.
**Fig. S5.** Student responses following completion of health and safety card games stratified on their ethnic status.
**Table S1.** The overall feedback survey provided to students upon completion of the Health and Safety Card Games.
**Table S2.** The pre and post feedback survey provided to students to evaluate the health and safety card games.

## Data Availability

All anonymised data analysed as part of this manuscript is available from the corresponding author upon reasonable request. Requests to access the datasets should be directed to the corresponding author: Dr Matthew Jones, E‐mail: m.a.jones9@salford.ac.uk.
