# Supplementary material for: Card games are effective tools to enhance foundation year health and safety inductions
Source: FEBS Open Bio. 2025 Oct 13;15(12):2080–95. doi: 10.1002/2211-5463.70140 (PMC12667211; doi:10.1002/2211-5463.70140)
Supplement: Supplementary file 1 — Fig. S1. Student responses following completion of health and safety card games stratified on their likelihood to participate in higher education (TUNDRA LSOA). Fig. S2. Student responses following completion of health and safety card games stratified on their likelihood to participate in higher education (ADULT HE). Fig. S3. Student responses following completion of health and safety card games stratified on their higher education entry qualification. Fig. S4. Student responses following completion of health and safety card games stratified on their identified gender. Fig. S5. Student responses following completion of health and safety card games stratified on their ethnic status. Table S1. The overall feedback survey provided to students upon completion of the Health and Safety Card Games. Table S2. The pre and post feedback survey provided to students to evaluate the health and safety card games. [file FEB4-15-2080-s001.docx]

**Card games are effective tools to enhance foundation year health and safety inductions**

***Authors’ names and affiliations*.**

Rafiq R.^1,2^, Matthews H.^1,2^, Abedalreza D.^2^, Yahya F.^2^, Jones M.A.^1,2^*

1. Biomedical Research Centre, University of Salford, Manchester, United Kingdom.
2. School of Science, Engineering and Environment, University of Salford, Manchester, United Kingdom.

*** Corresponding Author**

Dr Matthew Jones

**Address:** Room G45, Peel Building, Peel Park Campus, University of Salford, Manchester, United Kingdom, M5 4WT.

**Email:** [M.A.Jones9@salford.ac.uk](mailto:M.A.Jones9@salford.ac.uk)

**Phone:** +44 0161 295 2037

***Keywords:*** Higher Education; Card Games; Health and Safety; Foundation Year; Laboratory Education; Playful Learning.

***Abbreviations:*** Analysis of variance (ANOVA); Control of substances hazardous to health (COSHH); Global Harmonised System of Classification (GHS); Health and care professions council (HCPC); Health and safety (H&S); Higher Education (HE); Personal Protective Equipment (PPE); Tracking underrepresentation by area lower super output area (TUNDRA LSOA).

***Running heading:***  Card games enhance health and safety inductions

# **Supplementary Material**

**Supplementary Table 1. The overall feedback survey provided to students upon completion of the Health and Safety Card Games.**

| **Question 1** | Do you agree to participate in this research project? |
| --- | --- |
|  | **☐ Yes ☐ No** |
| **Question 2** | Please provide describe the gender you identify as |
|  | **☐ Male ☐ Female ☐ Non-Binary ☐ Other ☐ Prefer not to say** |
| **Question 3** | Please state your age at the time of survey completion |
|  | **Open Answer Question** |
| **Question 4** | Please describe your ethnic background (e.g. Black British, British Indian, Nigerian etc) |
|  | **Open Answer Question** |
| **Question 5** | Are you classified as a home (UK based) or international student? |
|  | **☐ Home ☐ International** |
| **Question 6** | Please provide your home (none-university) postcode. This will allow us to determine the socioeconomic status of the area you are from and the likelihood of people from this area attending university. |
|  | **Open Answer Question** |
| **Question 7** | What University entry qualification did you conduct before joining the University of Salford? |
|  | **☐ A-level ☐ BTEC ☐ Access Course ☐ International Qualification ☐ Other** |
| **Question 8** | Which Foundation Year programme are you currently enrolled on? |
|  | **☐ Biomedical Science ☐ Human Biology ☐ Biological Science ☐ Biochemistry ☐ Pharmaceutical Science ☐ Chemistry ☐ Marine Biology ☐ Wildlife** **Conservation with zoo biology ☐ Wildlife Conservation ☐ Other** |
| **Question 9** | What sort of career are you interested in pursuing post-graduation? |
|  | **Open Answer Question** |
| ***On a scale of 1 (strongly disagree) to 5 (strongly agree), please rate the following statements*** | |
| **Question 10** | *“I liked taking part in the session”* |
|  | ***Scale of 1 (strongly disagree) to 5 (strongly agree)*** |
| **Question 11** | *“I learnt a lot from participating in this session”* |
|  | ***Scale of 1 (strongly disagree) to 5 (strongly agree)*** |
| **Question 12** | *“I feel the session supported the development of my team working skills”* |
|  | ***Scale of 1 (strongly disagree) to 5 (strongly agree)*** |
| **Question 13** | *"This session allowed me to develop my communication skills"* |
|  | ***Scale of 1 (strongly disagree) to 5 (strongly agree)*** |
| **Question 14** | *“I feel the session supported the development of my confidence in laboratory health and safety”* |
|  | ***Scale of 1 (strongly disagree) to 5 (strongly agree)*** |
| **Question 15** | *“I feel the session supported the development of key employability skills”* |
|  | ***Scale of 1 (strongly disagree) to 5 (strongly agree)*** |
| **Question 16** | *"This session helped me better understand laboratory health and safety"* |
|  | ***Scale of 1 (strongly disagree) to 5 (strongly agree)*** |
| **Question 17** | *"This session gave me a better idea of the personal protective equipment used in laboratories"* |
|  | ***Scale of 1 (strongly disagree) to 5 (strongly agree)*** |
| **Question 18** | *"This session will help me with my upcoming practical classes/studies"* |
|  | ***Scale of 1 (strongly disagree) to 5 (strongly agree)*** |
| **Question 19** | *"This session was a valuable addition to my health and safety induction"* |
|  | ***Scale of 1 (strongly disagree) to 5 (strongly agree)*** |
| ***Game specific surveys. On a scale of 1 (strongly disagree) to 5 (strongly agree), please rate the following statements. Questions were repeated for each game.*** | |
| **Question 20** | *“This game was fun to play”* |
|  | ***Scale of 1 (strongly disagree) to 5 (strongly agree)*** |
| **Question 21** | *“This game increased my knowledge of laboratory health and safety”* |
|  | ***Scale of 1 (strongly disagree) to 5 (strongly agree)*** |
| **Question 22** | *“The instructions for this game were clear”* |
|  | ***Scale of 1 (strongly disagree) to 5 (strongly agree)*** |
| **Question 23** | *"* *The game was well designed"* |
|  | ***Scale of 1 (strongly disagree) to 5 (strongly agree)*** |
| **Question 24** | *“The game was visually appealing”* |
|  | ***Scale of 1 (strongly disagree) to 5 (strongly agree)*** |
| **Question 25** | *“I would recommend this game to other students to enhance their health and safety knowledge”* |
|  | ***Scale of 1 (strongly disagree) to 5 (strongly agree)*** |
| **Question 26** | *“This game increased my knowledge of personal protective equipment” (Asked for Personal Protective Equipment (PPE) Lab Gear Up Only)* |
|  | ***Scale of 1 (strongly disagree) to 5 (strongly agree)*** |
| **Question 27** | *“This game increased my knowledge of laboratory equipment” (Asked for Lab Equipment Identifier Only)* |
|  | ***Scale of 1 (strongly disagree) to 5 (strongly agree)*** |

**Supplementary Table 2. The pre and post feedback survey provided to students to evaluate the health and safety card games.**

| **Pre-Questions** | |
| --- | --- |
| **Pre-Question 1** | *On a scale of 1 (Least)-10 (Most), please rate your knowledge of laboratory health and safety?* |
|  | **Rating scale of 1 to 10** |
| **Pre-Question 2** | *On a scale of 1-10, please rate your knowledge of what Personal protective equipment is?* |
|  | **Rating scale of 1 to 10** |
| **Pre-Question 3** | *On a scale of 1-10, please rate your knowledge of health and safety symbols?* |
|  | **Rating scale of 1 to 10** |
| **Pre-Question 4** | *On a scale of 1-10, please rate your knowledge of when Personal protective equipment should be worn in laboratories?* |
|  | **Rating scale of 1 to 10** |
| **Pre-Question 5** | *On a scale of 1-10, please rate your knowledge of identifying laboratory hazards and risks?* |
|  | **Rating scale of 1 to 10** |
| **Pre-Question 6** | *On a scale of 1-10, please rate your knowledge of identifying laboratory equipment?* |
|  | **Rating scale of 1 to 10** |
| **Post-Questions** | |
| **Post-Question 1** | *On a scale of 1 (Least)-10 (Most), please rate your knowledge of laboratory health and safety?* |
|  | **Rating scale of 1 to 10** |
| **Post-Question 2** | *On a scale of 1-10, please rate your knowledge of what Personal protective equipment is?* |
|  | **Rating scale of 1 to 10** |
| **Post-Question 3** | *On a scale of 1-10, please rate your knowledge of health and safety symbols?* |
|  | **Rating scale of 1 to 10** |
| **Post-Question 4** | *On a scale of 1-10, please rate your knowledge of when Personal protective equipment should be worn in laboratories?* |
|  | **Rating scale of 1 to 10** |
| **Post-Question 5** | *On a scale of 1-10, please rate your knowledge of identifying laboratory hazards and risks?* |
|  | **Rating scale of 1 to 10** |
| **Post-Question 6** | *On a scale of 1-10, please rate your knowledge of identifying laboratory equipment?* |
|  | **Rating scale of 1 to 10** |

**
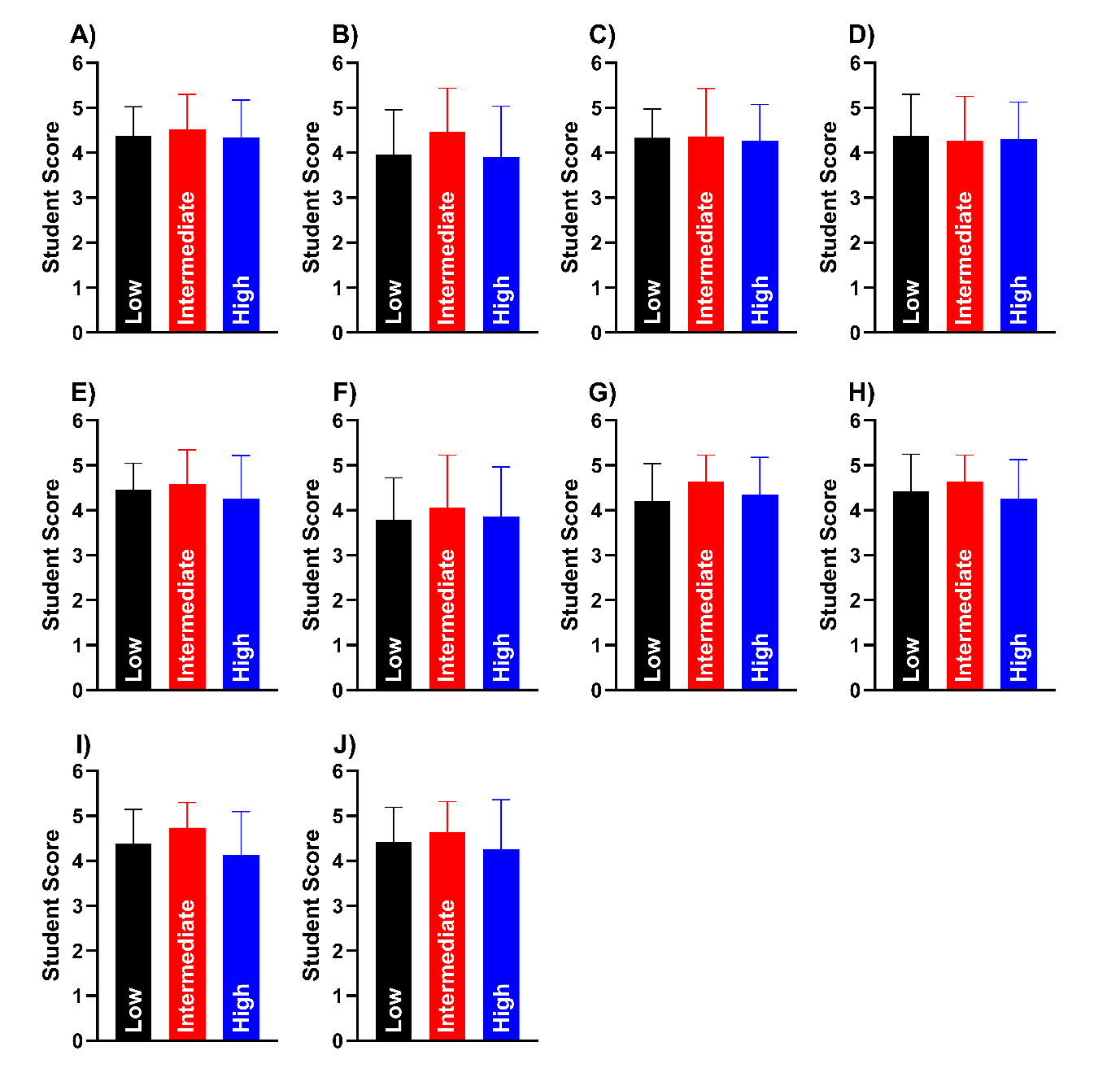
Supplementary Figure 1. Student responses following completion of health and safety card games stratified on their likelihood to participate in higher education (TUNDRA LSOA).** Student questions relating to **A)** enjoyment, **B)** learning, **C)** teamwork development, **D)** communication development, **E)** health and safety confidence, **F)** employability development, **G)** health and safety understanding, **H)** personal protective equipment knowledge, **I)** support upcoming classes, and **J)** valuable addition to laboratory health and safety inductions were stratified. Black bars indicate student scores from low TUNDRA LSOA areas (TUNDRA LSOA scores of 1 or 2) (n = 24), red bars indicate student scores from intermediate TUNDRA LSOA areas (TUNDRA LSOA score of 3) (n = 19), and blue bars indicate student scores from high TUNDRA LSOA areas (TUNDRA LSOA scores of 4 or 5) (n = 23). * indicates statistical significance to a *P* value of < 0.05 following a one-way ANOVA with a Tukey post-hoc test. All data expressed as mean ± standard deviation.


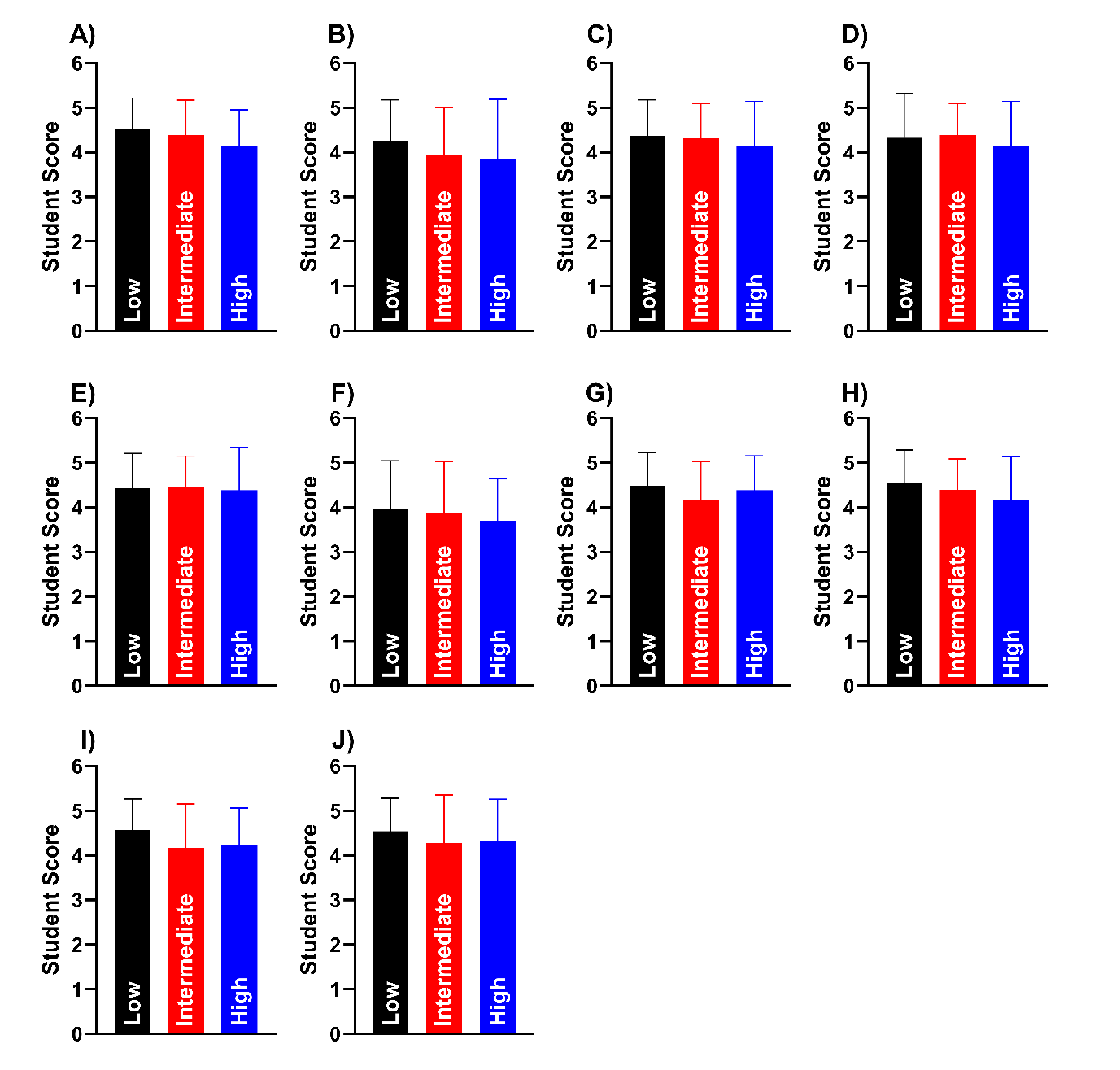
**Supplementary Figure 2. Student responses following completion of health and safety card games stratified on their likelihood to participate in higher education (ADULT HE).** Student questions relating to **A)** enjoyment, **B)** learning, **C)** teamwork development, **D)** communication development, **E)** health and safety confidence, **F)** employability development, **G)** health and safety understanding, **H)** personal protective equipment knowledge, **I)** support upcoming classes, and **J)** valuable addition to laboratory health and safety inductions were stratified. Black bars indicate student scores from low ADULTHE areas (ADULTHE scores of 1 or 2) (n = 35), red bars indicate student scores from intermediate ADULTHE areas (ADULTHE score of 3) (n = 18), and blue bars indicate student scores from high ADULTHE areas (ADULTHE scores of 4 or 5) (n = 13). * indicates statistical significance to a *P* value of < 0.05 following a one-way ANOVA with a Tukey post-hoc test. All data expressed as mean ± standard deviation.

**
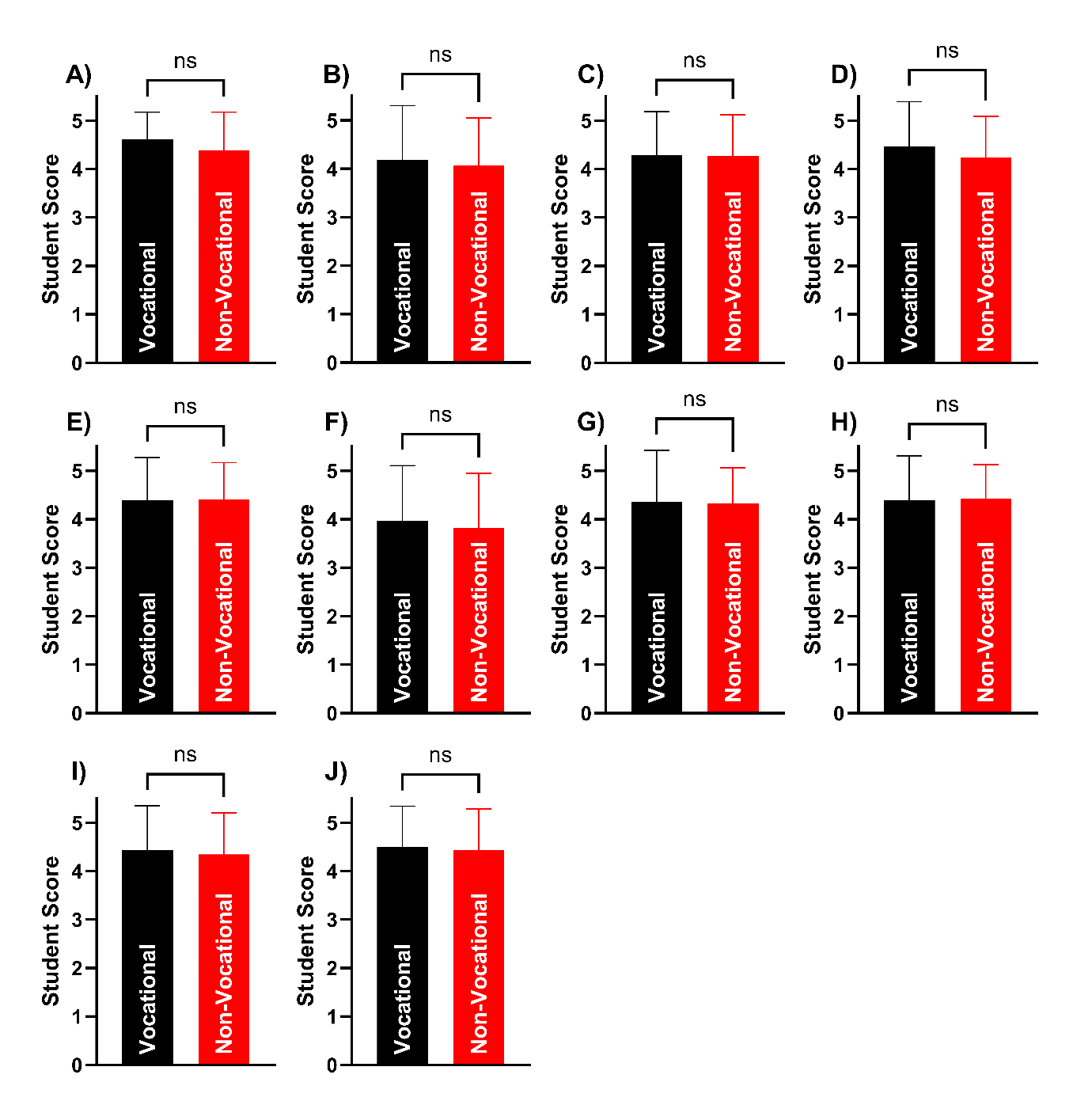
**

**Supplementary Figure 3. Student responses following completion of health and safety card games stratified on their higher education entry qualification.** Student questions relating to **A)** enjoyment, **B)** learning, **C)** teamwork development, **D)** communication development, **E)** health and safety confidence, **F)** employability development, **G)** health and safety understanding, **H)** personal protective equipment knowledge, **I)** support upcoming classes, and **J)** valuable addition to laboratory health and safety inductions were stratified. Black bars indicate student scores from those who previously studied vocational qualifications (n = 28), and red bars indicate student scores from those who previously studied non-vocational qualifications areas (n = 44). * indicates statistical significance to a *P* value of < 0.05 following unpaired t-tests. All data expressed as mean ± standard deviation.

**
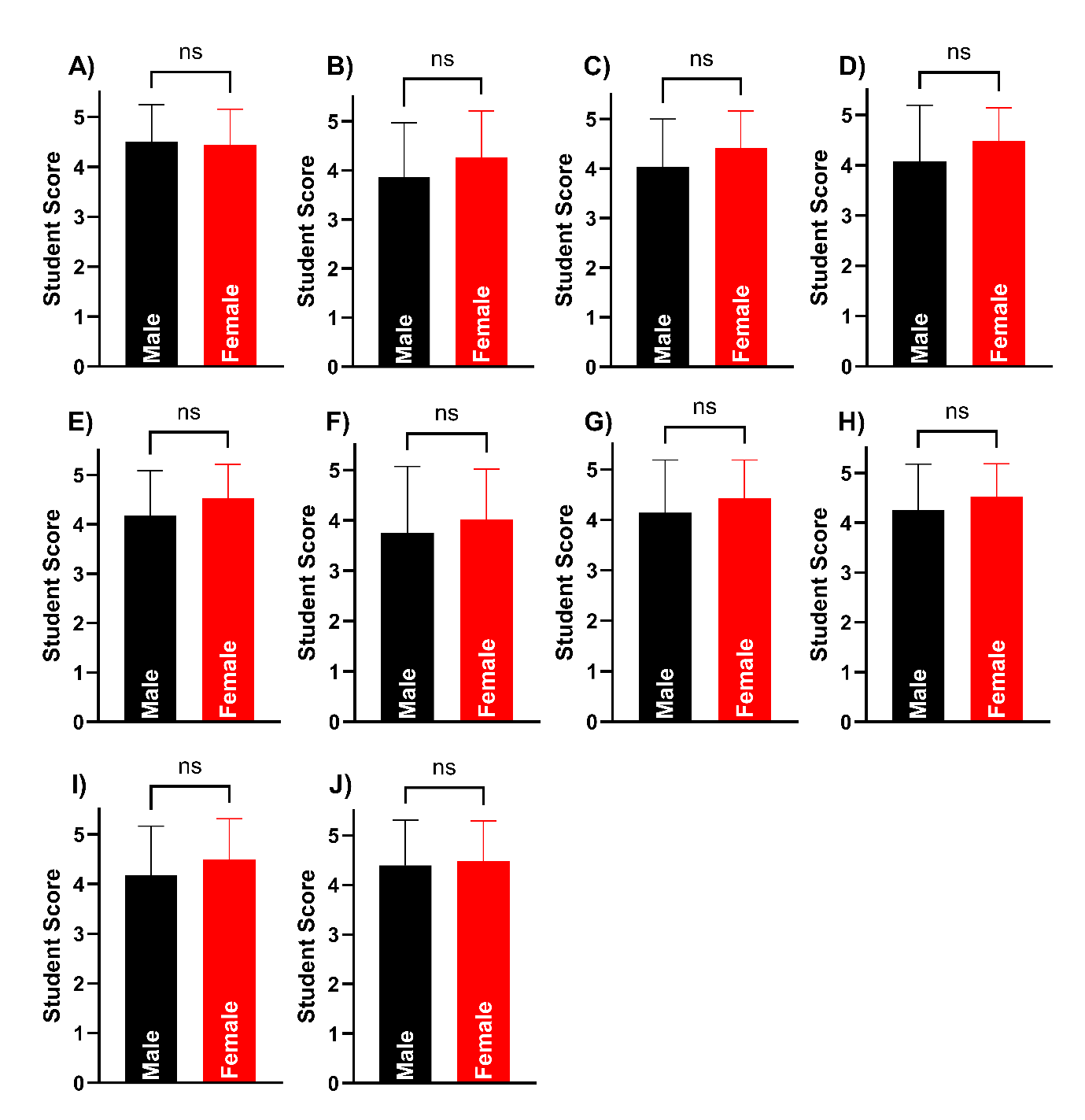
**

**Supplementary Figure 4. Student responses following completion of health and safety card games stratified on their identified gender.** Student questions relating to **A)** enjoyment, **B)** learning, **C)** teamwork development, **D)** communication development, **E)** health and safety confidence, **F)** employability development, **G)** health and safety understanding, **H)** personal protective equipment knowledge, **I)** support upcoming classes, and **J)** valuable addition to laboratory health and safety inductions were stratified. Black bars indicate student scores from those who identified as male (n = 28), and red bars indicate student scores from those who identified as female (n = 46). All data expressed as mean ± standard deviation.

**
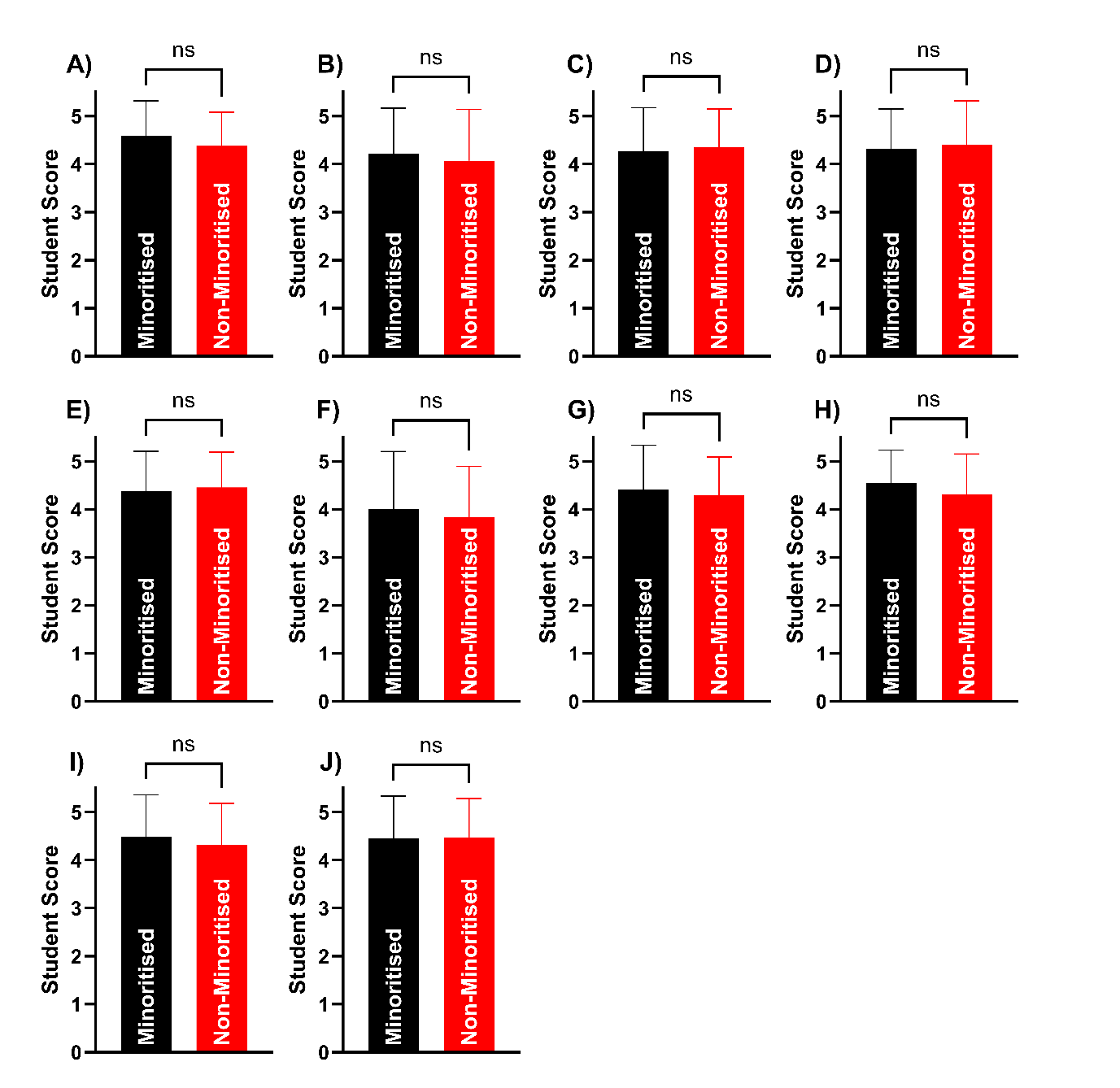
**

**Supplementary Figure 5. Student responses following completion of health and safety card games stratified on their ethnic status.** Student questions relating to **A)** enjoyment, **B)** learning, **C)** teamwork development, **D)** communication development, **E)** health and safety confidence, **F)** employability development, **G)** health and safety understanding, **H)** personal protective equipment knowledge, **I)** support upcoming classes, and **J)** valuable addition to laboratory health and safety inductions were stratified. Black bars indicate student scores from who reported being from a minoritised background (n = 37), and red bars indicate student scores from who reported being from a minoritised background (n = 42). All data expressed as mean ± standard deviation.
